# Production performance and rumen bacterial community structure of *Hu* sheep fed fermented spent mushroom substrate from *Pleurotus eryngii*

**DOI:** 10.1038/s41598-023-35828-8

**Published:** 2023-05-29

**Authors:** Xiaoyun Huang, Liuting Zhou, Xiaofeng You, Haidong Han, Xinzhu Chen, Xiusheng Huang

**Affiliations:** 1 Agriculture Ecology Research Institute, Fujian Academy of Agricultural Sciences, Fuzhou, 350013 China; 2 Fujian Engineering and Technology Research Center for Recycling Agriculture in Hilly Areas, Fuzhou, 350013 China; 3 Institute of Animal Husbandry and Veterinary Medicine, Fujian Academy of Agricultural Sciences, Fuzhou, 350013 China

**Keywords:** Agroecology, Microbial ecology, Bacteria, Microbial communities

## Abstract

This study aimed to investigate the effect of fermented spent mushroom substrate from *Pleurotus eryngii* (*SMPE*) supplementation on production performance, meat quality and rumen bacterial community structure of *Hu* sheep. 120 2-month-old *Hu* sheep with average body weight [(13.50 ± 3.10) kg] were selected and randomly divided into 4 groups with 3 replicates per group and 10 sheep per replicate. The control group (RL1) was fed a total mixed ration (*TMR*), and group RL2, RL3 and RL4 were fed the basal diets supplemented with 15%, 30% and 45% fermented SMPE, respectively. The pretest period lasted for 10 days and the test period lasted for 150 days. The results showed that: (1) Difference (*p* < 0.05) was observed in average daily feed intake (*ADFI*) and feed conversion ratio (*FCR*) between RL2 and RL4 groups. The eye muscle area (*EMA*) and grade rule (*GR*) values in RL2 and RL3 were significantly higher than those in RL1 and RL4 groups (*p* < 0.05). (2) The contents of threonine, valerine, leucine, lysine, histidine, essential amino acids, flavor amino acids, aspartic acid, serine, glutamic acid and arginine of the *longissimus dorsi* muscle in RL2 and RL3 groups were significantly higher than RL1 and RL4 (*p* < 0.05). (3) A total of 1,202,445 valid sequences were obtained from rumen of *Hu* sheep fed different amounts of fermented feed, and the valid sequences were clustered into 9824 Operational Taxonomic Units (OTUs). (4) α diversity analysis showed that the richness and diversity of rumen bacterial communities in *Hu* sheep in RL1, RL2, RL3 and RL4 groups were significantly higher than RL0 (raw materials of fermented SMPE) group (*p* < 0.05). *β* diversity analysis showed that the bacterial community structure was the most different between RL0 and RL3. (5) At the genus level, compared with RL1, the relative abundance of *Christensenellaceae* R-7 in RL3 group decreased significantly by 33.59%, the relative abundance of *Prevotellaceae* UCG001 in RL2, RL3 and RL4 decreased significantly by 50.41%, 62.24% and 49.17%, respectively, and the relative abundance of *Ruminococcaceae* NK4A214 in RL2 group increased significantly by 35.01% (*p* < 0.05). In summary, the addition of fermented SMPE to TMR can significantly improve the production performance, meat quality and rumen bacterial community diversity and abundance of *Hu* sheep.

## Introduction

The lack of feed raw materials and their cost have always been an important factor restricting the development of animal husbandry industry^1^. In recent years, the development and utilization of new feed resources has become an urgent problem to be solved. China is a major producer of edible fungi, ranking first in the world in annual output^2^. *Pleurotus eryngii*, commonly called the King Oyster mushroom, is a valuable edible mushroom among consumers owing to the unique flavor and high nutritional values^3^. With the large-scale and intensive production of *Pleurotus eryngii*, thus caused a large quantity of spent mushroom substrate from *Pleurotus eryngii* (*SMPE*) that is not utilized effectively.

SMPE is the remaining media residue after harvesting, which has a wide range of sources and low price. SMPE has shown great potential as animal feed, its protein content and amino acid composition are close to corn, and the crude fiber content is close to roughage, which is an excellent “neutral” feed^4^. Studies have shown that spent mushroom substrate is rich in crude fiber, crude protein, polysaccharides, crude fat, calcium, phosphorus and other active nutrients, and is a new type of high-quality feed raw material for livestock and poultry^5,6^. However, spent mushroom substrate is not fully utilized in the actual production process at present, only a very small part is developed for animal feed, most of them are directly incinerated or discarded as waste, which not only wastes biological resources but also causes serious environmental pollution problems. Therefore, the rational development utilization technology of spent mushroom substrate as feed can not only turn waste into treasure and protect the environment, but also alleviate the problem of shortage of feed resources and improve the economic benefits of animal husbandry industry, which has important ecological significance and broad market prospects.

The main components of SMPE are agricultural byproducts of high fibre raw materials such as bagasse, wheat bran, corn flour, etc*.*, resulting in low nutritional value, poor palatability, low digestion and utilization^7,8^. In addition, SMPE are prone to mildew or breeding pathogenic bacteria due to the high content of moisture, loose and porous raw material characteristics^9^. However, the nutritional value and palatability of SMPE can be improved through microbial fermentation. After microbial fermentation, macromolecular substances such as cellulose and hemicellulose are degraded into carbohydrates, amino acids, vitamins and other nutrients that are easily digested and absorbed by the animal, improving palatability and prolonging the preservation time. Lactic acid bacteria (*LAB*) and yeast are the most commonly used probiotics, they can not only convert the fermentation matrix into bacterial protein to improve the nutritional value, but also can produce flavor substances include acids, alcohols, esters and other aromatic substances to improve feed palatability^10^. LAB can produce organic acids, bacteriocins, hydrogen peroxide and other metabolites with bacteriostatic activity to inhibit the growth of other harmful bacteria^11^. *Saccharomyces cerevisiae* helps lactic acid bacteria due to use of substrates such as lactic acid and organic acids^12^. In addition, *Bacillus subtilis* has high protease and amylase activity^13^. Therefore, the construction of mixed-strain fermentation has been widely concerned.

Previous studies have shown that mushroom substrate feed is of great significance in the development and utilization of unconventional feed for ruminants^14^. Rumen is a unique digestive organ of ruminants, and there are a large number of microorganisms inside it, mainly including bacteria, fungi, archaea, protozoa, etc*.*, of which anaerobic bacteria are the dominant microorganisms^15^. Rumen bacteria are closely related to ruminant production performance and meat quality^16^. The feed is fermented and decomposed under the action of microorganisms after entering the rumen, which is conducive to the efficient absorption of nutrients by the animal. Henderson et al*.*^17^ and Maga et al.^18^ found that feed is the dominant factor affecting the change of rumen microbial community structure in ruminants, which in turn affects the digestion and absorption of nutrients and energy supply. Therefore, understanding the microbial community composition structure of the rumen is the key to promote feed digestion and absorption and improve animal production performance.

We hypothesized that the supplementation of fermented SMPE with appropriate proportion might positively affect *Hu* sheep productivity, meat quality and rumen bacterial community. To test this hypothesis, the objectives of the present study were to determine the effects of different amounts of fermented SMPE on the production performance, meat quality and rumen bacterial community structure of *Hu* sheep.

## Results

### Effects of fermented spent mushroom substrate on production performance and meat quality of *Hu* sheep

According to Table 1, ADFI of RL1 and RL2 groups was significantly higher than that of RL4 (*p* < 0.05). FCR of RL2 was the lowest and significantly different from RL3 and RL4 groups (*p* < 0.05), while RL4 group was significantly higher than other groups (*p* < 0.05). No difference (*p* > 0.05) was observed in IBW, FBW, WG and ADG between groups. The EMA and GR values in the RL2 and RL3 were significantly higher than those in the RL1 and RL4 (*p* < 0.05). Compared with RL1, the EMA of RL2 and RL3 increased by 37.23% and 42.30%, and the GR value increased by 60.00% and 66.67%, respectively. No difference (*p* > 0.05) was observed in PSW, CW, SR, ST and back fat thickness (BFT) between RL1 and RL2 (Table 2). In addition, there were no significant differences in MCP, pH of the *longissimus dorsi* muscle at 1 h and 24 h after slaughter, DR at 24 h and 48 h after slaughter (*p* > 0.05) (Table S.1). The results showed that the use of fermented SMPE as the diet of *Hu* sheep had no significant effect on the meat quality.

**Table 1:** Effects of different fermented *Pleurotus eryngii* mushroom substrate addition on growth performance of *Hu* sheep.

Treatment	IBW (kg)	FBW (kg)	WG (kg)	ADG (kg)	ADFI (kg)	FCR
RL1	13.78 ± 0.76	33.08 ± 1.03	19.30 ± 1.63	0.13 ± 0.01	1.48 ± 0.04^a^	11.56 ± 0.06^bc^
RL2	14.28 ± 1.44	34.27 ± 4.00	19.99 ± 3.26	0.13 ± 0.02	1.50 ± 0.03^a^	11.48 ± 0.05^c^
RL3	14.20 ± 1.56	32.85 ± 0.76	18.65 ± 1.82	0.12 ± 0.01	1.45 ± 0.02^ab^	11.71 ± 0.16^b^
RL4	13.77 ± 0.75	30.87 ± 0.92	17.10 ± 1.52	0.11 ± 0.01	1.40 ± 0.05^b^	12.08 ± 0.12^a^

RL1, RL2, RL3, RL4 represent groups of *Hu* sheep fed TMR diets contained fermented SMPE at 0%, 15%, 30% and 45%, respectively. IBW, initial body weight, FBW, final body weight, WG, weight gain, ADG, average daily gain, ADFI, average daily feed intake, FCR, feed conversion ratio. The different letter in each column indicates a significant difference (*p* < 0.05, n = 30).

**Table 2:** Effects of different fermented *Pleurotus eryngii* mushroom substrate addition on the slaughter performance of *Hu* sheep.

Treatment	PSW (kg)	CW (kg)	SR (%)	ST (cm)	BFT (cm)	EMA (cm^2^)	GR (cm)
RL1	36.80 ± 6.51	16.95 ± 3.39	45.96 ± 1.10	0.15 ± 0.01	0.13 ± 0.00	9.67 ± 0.05^b^	0.90 ± 0.08^b^
RL2	41.54 ± 1.47	19.78 ± 1.66	47.56 ± 2.32	0.18 ± 0.01	0.27 ± 0.09	13.27 ± 2.66^a^	1.44 ± 0.00^a^
RL3	39.73 ± 3.35	18.83 ± 1.59	47.38 ± 0.01	0.16 ± 0.03	0.21 ± 0.01	13.76 ± 3.78^a^	1.50 ± 0.32^a^
RL4	39.27 ± 3.01	18.47 ± 1.86	46.98 ± 1.13	0.18 ± 0.04	0.16 ± 0.07	9.38 ± 1.20^b^	1.06 ± 0.12^b^

RL1, RL2, RL3, RL4 represent groups of *Hu* sheep fed TMR diets contained fermented SMPE at 0%, 15%, 30% and 45%, respectively. PSW, pre-slaughter weight, CW, carcass weight, SR, slaughter rate, ST, skin thickness, BFT, back fat thickness, EMA, eye muscle area, GR, grade rule. The different letter in each column indicates a significant difference (*p* < 0.05, n = 3).

Among the essential amino acids (*EAAs*), the contents of threonine, valerine, leucine, lysine, histidine and total amount of essential amino acids of the *longissimus dorsi* muscle in RL2 and RL3 were significantly higher than those in RL1 and RL4 (*p* < 0.05) (Table 3), the content of isoleucine was significantly higher than RL4 (*p* < 0.05), and the contents of methionine and phenylalanine were not significantly different between samples (*p* > 0.05). Compared with RL1, the contents of threonine, valerine, leucine, lysine, histidine and total essential amino acids in the RL2 significantly increased by 20.81%, 16.17%, 21.04%, 24.49%, 17.37% and 20.35%, respectively, while the RL3 increased by 20.47%, 18.86%, 20.84%, 24.49%, 19.25% and 19.97%, respectively.

**Table 3:** Effects of different fermented *Pleurotus eryngii* mushroom substrate addition on amino acid content of mutton (g·kg^−1^).

Items	Groups			
	RL1	RL2	RL3	RL4
Essential amino acid				
Threonine	2.98 ± 0.40^b^	3.60 ± 0.05^a^	3.59 ± 0.09^a^	2.80 ± 0.03^b^
Valerine	3.34 ± 0.44^b^	3.88 ± 0.22^a^	3.97 ± 0.07^a^	3.27 ± 0.07^b^
Methionine	1.09 ± 0.41	1.54 ± 0.25	1.32 ± 0.12	1.24 ± 0.58
Isoleucine	2.93 ± 0.43^bc^	3.45 ± 0.10^ab^	3.47 ± 0.11^a^	2.55 ± 0.32^c^
Leucine	4.99 ± 0.70^b^	6.04 ± 0.11^a^	6.03 ± 0.10^a^	4.66 ± 0.20^b^
Phenylalanine	3.28 ± 0.50	3.72 ± 0.34	3.72 ± 0.35	3.07 ± 0.08
Lysine	5.35 ± 0.81^b^	6.66 ± 0.10^a^	6.66 ± 0.11^a^	5.16 ± 0.16^b^
Histidine	2.13 ± 0.37^b^	2.50 ± 0.08^a^	2.54 ± 0.07^a^	2.03 ± 0.04^b^
EAAs	26.09 ± 3.95^b^	31.40 ± 0.82^a^	31.30 ± 0.58^a^	24.78 ± 0.71^b^
Non-essential amino acids				
Aspartic acid	6.01 ± 0.76^b^	7.13 ± 0.07^a^	7.08 ± 0.09^a^	5.56 ± 0.22^b^
Serine	2.56 ± 0.29^b^	3.15 ± 0.13^a^	2.95 ± 0.10^a^	2.42 ± 0.04^b^
Glutamic acid	10.58 ± 1.21^b^	12.46 ± 0.07^a^	12.43 ± 0.11^a^	10.08 ± 0.12^b^
Glycine	5.08 ± 0.19^a^	4.27 ± 0.20^c^	4.34 ± 0.22^bc^	4.90 ± 0.51^ab^
Alanine	4.52 ± 0.34^ab^	4.84 ± 0.26^a^	4.76 ± 0.13^ab^	4.35 ± 0.16^b^
Tyrosine	2.09 ± 0.33^ab^	2.47 ± 0.25^a^	2.42 ± 0.16^a^	1.93 ± 0.11^b^
Arginine	4.32 ± 0.41^b^	5.31 ± 0.24^a^	5.24 ± 0.29^a^	4.24 ± 0.21^b^
Proline	3.56 ± 0.11^a^	3.26 ± 0.07^ab^	3.21 ± 0.13^b^	3.36 ± 0.28^ab^
FAAs	30.51 ± 2.51^b^	34.01 ± 0.51^a^	33.85 ± 0.35^a^	29.13 ± 0.77^b^

RL1, RL2, RL3, RL4 represent groups of *Hu* sheep fed TMR diets contained fermented SMPE at 0%, 15%, 30% and 45%, respectively. EAAs, essential amino acids, FAAs, flavor amino acids. The different letter in each line indicates a significant difference (*p* < 0.05, n = 3).

Among the non-essential amino acids, the contents of aspartic acid, serine, glutamic acid and arginine of the *longissimus dorsi* muscle in RL2 and RL3 were significantly higher than those in RL1 and RL4 (*p* < 0.05), and the tyrosine content was significantly higher than that in the RL4 (*p* < 0.05). Compared with RL1, the contents of aspartic acid, serine, glutamic acid and arginine in RL2 were significantly increased by 18.64%, 23.05%, 17.77% and 22.92%, respectively, and by 17.80%, 15.23%, 17.49% and 21.30% in RL3, respectively. For flavor amino acids (*FAAs*), the contents in RL2 and RL3 were significantly higher than those in RL1 and RL4 (*p* < 0.05).

### OTU composition and structure of bacterial community

**Figure 1:**
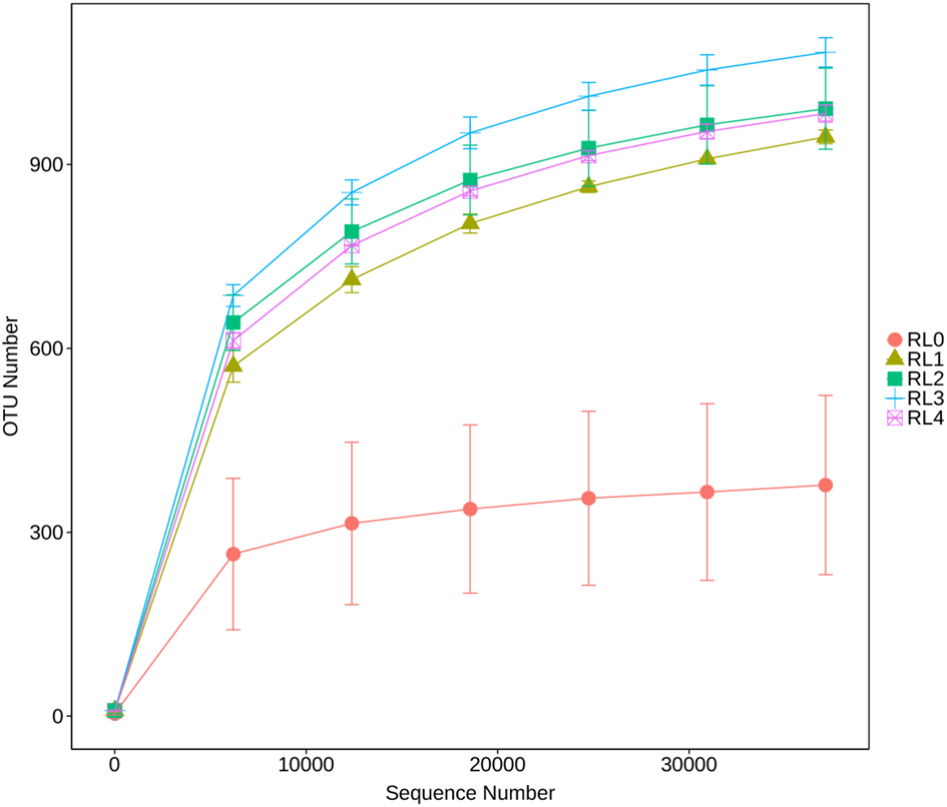
Dilution curves of rumen bacterial community of *Hu* sheep fed with different fermented *Pleurotus eryngii* mushroom substrate addition. RL0 represent raw materials of fermented SMPE, RL1, RL2, RL3, RL4 represent rumen liquid of *Hu* sheep fed TMR diets contained fermented SMPE at 0%, 15%, 30% and 45%, respectively.

After quality control of data obtained by the IonS5^TM^XL sequencing platform, a total of 1,202,445 valid sequences were obtained from raw materials and rumen liquid of *Hu* sheep, with an average valid sequences of 80,163 per sample. Valid sequences were clustered as 9824 OTUs at a sequence similarity threshold of 97%. Among them, RL0 had an average of 417 OTUs, RL1 was 980, RL2 was 1081, RL3 was 1165, and RL4 was 1061. The number of OTUs in RL0 was significantly lower than others (*p* < 0.05). The dilution curve directly reflects whether the extracted optimized sequence depth is reasonable and indirectly reflects the species richness in the sample. It can be seen from Fig. 1 that the number of sequences extracted reaches more than 30,000, and the curves tend to be flat, indicated that the sequence reservoir volume measured by different samples can better reflect the number of bacterial community species, and the amount of sequencing data was basically reasonable.

**Figure 2:**
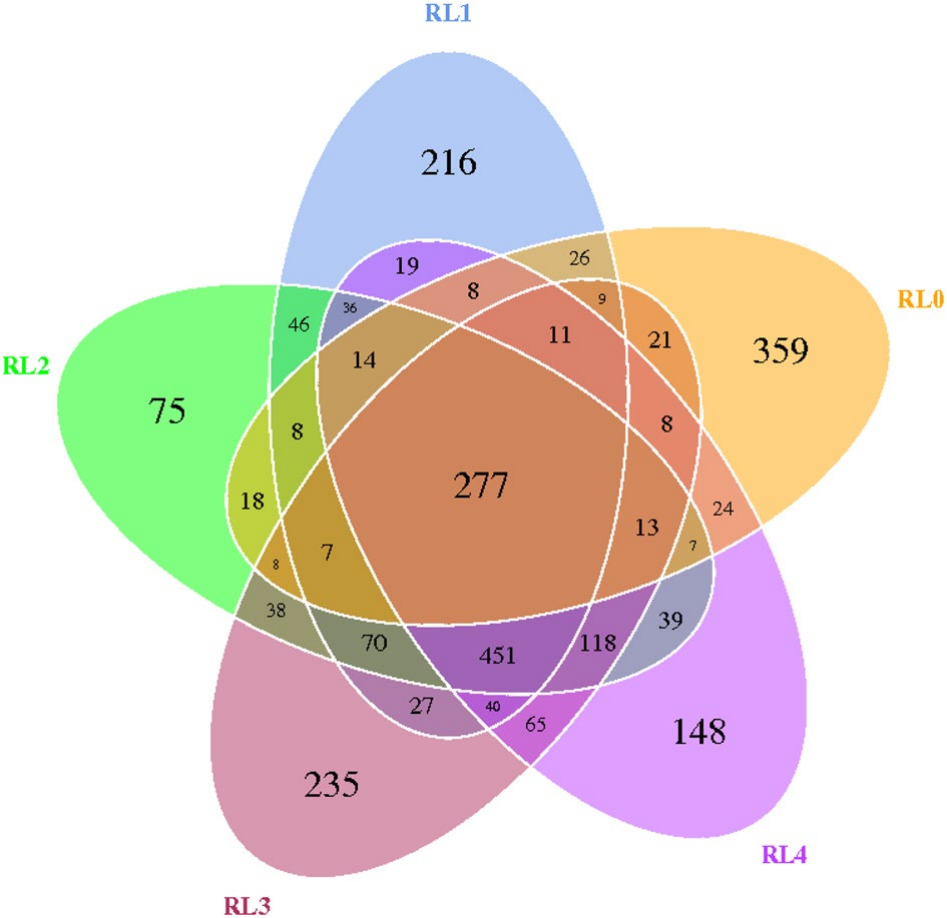
Venn diagram of rumen bacterial community of *Hu* sheep fed with different fermented *Pleurotus eryngii* mushroom substrate addition. RL0 represent raw materials of fermented SMPE, RL1, RL2, RL3, RL4 represent rumen liquid of *Hu* sheep fed TMR diets contained fermented SMPE at 0%, 15%, 30% and 45%, respectively.

The Venn diagram can visualize the differences and overlaps in the OTU composition of bacterial communities in different samples (Fig. 2). The results of Venn analysis showed that at the OTU level, specific bacterial OTU accounted for 14.71% (359) of the total OTU sequence number in RL0, specific bacterial OTU accounted for 8.85% (216) of the total OTU sequence number in RL1, specific bacterial OTU in RL2 accounted for 3.07% (75) of the total OTU sequence number, and specific bacterial OTU in RL3 accounted for 9.63% (235) of the total OTU sequence number. Specific bacterial OTUs in RL4 accounted for 6.06% (148) of the total OTU sequence number. In addition, the number of bacterial OTUs shared by RL0, RL1, RL2, RL3, and RL4 was 277 (11.35%).

### Analysis of bacterial community diversity

The species richness and uniformity of rumen bacterial communities treated with different amounts of fermented spent mushroom substrate were evaluated by *α* diversity index in the samples (cutoff = 37,136). Table S.2 showed that *α* diversity index of rumen bacterial community in RL1, RL2, RL3 and RL4 was significantly higher than RL0 (*p* < 0.05), indicated that the species diversity of rumen bacterial community was significantly higher than raw materials. However, with the increase of the addition of fermented SMPE, the differences in Observed species index, Shannon index, Simpson index, Chao1 index and ACE index were not significant (*p* > 0.05).

**Figure 3:**
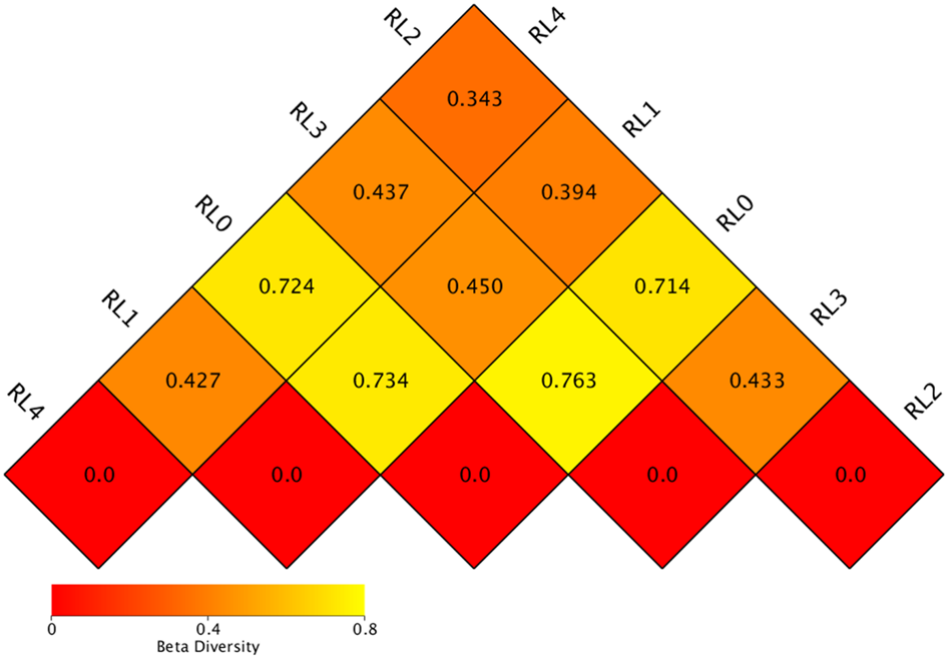
The β diversity index of rumen bacterial community of *Hu* sheep fed with different fermented *Pleurotus eryngii* mushroom substrate addition. RL0 represent raw materials of fermented SMPE, RL1, RL2, RL3, RL4 represent rumen liquid of *Hu* sheep fed TMR diets contained fermented SMPE at 0%, 15%, 30% and 45%, respectively.

The β diversity index could measure the degree of divergence in species diversity between two samples by Unweighted Unifrac distance. The smaller the value, the smaller the difference in species diversity between two samples. As can be seen from Fig. 3, the smallest distances of Unweighted Unifrac were RL2 and RL4, with a value of 0.343, and the largest distances were RL0 and RL3, with a value of 0.763. It can be seen that the difference in bacterial community structure between RL2 and RL4 was the smallest, and the difference between RL0 and RL3 was the largest.

### PCoA analysis of bacterial community

**Figure 4:**
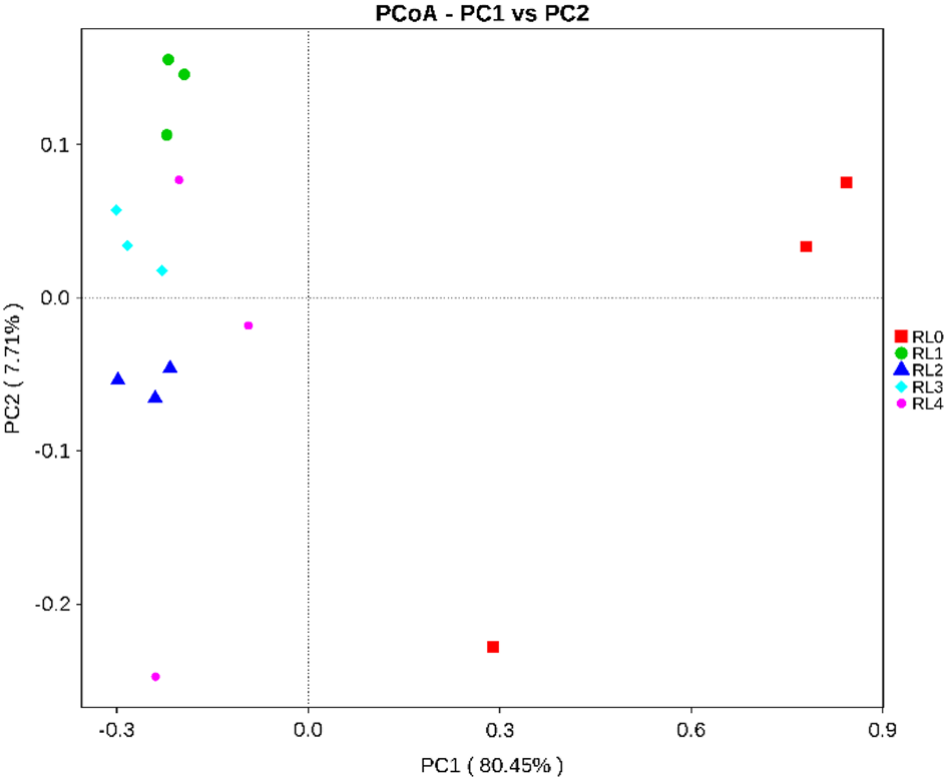
PCoA analysis of rumen bacterial community of *Hu* sheep fed with different fermented *Pleurotus eryngii* mushroom substrate addition. RL0 represent raw materials of fermented SMPE, RL1, RL2, RL3, RL4 represent rumen liquid of *Hu* sheep fed TMR diets contained fermented SMPE at 0%, 15%, 30% and 45%, respectively.

The results of Principal Co-ordinates Analysis (PCoA) of rumen bacterial community with different treatments based on OTU were shown in Fig. 4, and principal component 1 (PC1) and principal component 2 (PC2) explained 80.45% and 7.71% of the variance of the variables, respectively, and the cumulative contribution rate was 88.16%. PC1 clearly distinguished the bacterial community in RL0 from RL1, RL2, RL3 and RL4, and PC2 clearly distinguished between four groups, indicated that there were great differences in the bacterial community structure of raw materials and the rumen liquid of *Hu* sheep with different treatments.

### Changes in bacterial community composition and structure

A total of 10 phyla were detected in raw materials and rumen liquid of *Hu* sheep treated with different amounts of fermentation feed, the dominant taxa were as follows: Firmicutes (42.86–78.73%), Bacteroidetes (8.54–48.56%), Proteobacteria (0.71–7.49%) and Fibrobacteres (0.18–6.86%) (Figure S.1). Compared with RL1, the relative abundance of Firmicutes in the RL4 was significantly increased by 9.36% (*p* < 0.05), the relative abundance of Bacteroidetes in the RL3 was increased by 2.10%, but the difference was not significant (*p* > 0.05), and the relative abundance of Fibrobacteres in the RL4 was significantly increased by 68.24% (*p* < 0.05).

At the genus level, the dominant genera (abundance > 1%) of bacterial community of the RL0 were *Lactobacillus*, *Prevotella* 1, and *Bacteroides*. In the rumen of *Hu* sheep treated with different amounts of bran, *Prevotella* 1, *Christensenellaceae* R-7, *Ruminococcaceae* NK4A214, *Fibrobacter*, *Rikenellaceae* RC9, *Saccharofermentans* and *Prevotellaceae* UCG001 were all dominant genera in RL1, RL2, RL3 and RL4 (Table 4).

**Table 4:** Relative abundance of rumen bacterial community of *Hu* sheep fed with different fermented *Pleurotus eryngii* mushroom substrate addition based on genus level.

Genus	Relative abundance (%)				
	RL0	RL1	RL2	RL3	RL4
*Lactobacillus*	64.88 ± 36.24^a^	0.10 ± 0.01^b^	0.05 ± 0.00^b^	0.09 ± 0.00^b^	0.10 ± 0.02^b^
*Prevotella* 1	2.64 ± 4.41^b^	22.71 ± 3.46^a^	21.48 ± 2.94^a^	18.78 ± 9.13^a^	19.88 ± 2.79^a^
*Christensenellaceae* R-7	0.51 ± 0.68^c^	16.67 ± 3.19^a^	14.60 ± 3.33^ab^	11.07 ± 3.24^b^	16.26 ± 1.75^a^
*Ruminococcaceae* NK4A214	0.12 ± 0.16^c^	5.97 ± 0.85^b^	8.06 ± 1.01^a^	6.13 ± 1.12^b^	6.51 ± 1.37^ab^
*Fibrobacter*	0.18 ± 0.29^b^	4.15 ± 3.10^a^	5.39 ± 0.95^a^	5.15 ± 1.07^a^	6.94 ± 0.33^a^
*Rikenellaceae* RC9	0.33 ± 0.50^b^	5.09 ± 0.16^a^	5.60 ± 1.02^a^	6.32 ± 0.78^a^	4.92 ± 1.43^a^
*Saccharofermentans*	0.08 ± 0.12^c^	2.95 ± 0.92^ab^	1.56 ± 0.20^bc^	3.08 ± 0.73^ab^	3.83 ± 1.65^a^
*Prevotellaceae* UCG001	0.10 ± 0.11^c^	4.82 ± 0.59^a^	2.39 ± 0.31^b^	1.82 ± 0.38^b^	2.45 ± 0.89^b^
*Bacteroides*	2.41 ± 1.17^a^	0.03 ± 0.01^b^	0.01 ± 0.01^b^	0.01 ± 0.00^b^	0.02 ± 0.01^b^
*Ruminococcus*	0.13 ± 0.19^b^	0.65 ± 0.72^ab^	0.69 ± 0.50^ab^	0.86 ± 0.11^ab^	1.74 ± 1.38^a^

RL0 represent raw materials of fermented SMPE, RL1, RL2, RL3, RL4 represent rumen liquid of Hu sheep fed TMR diets contained fermented SMPE at 0%, 15%, 30% and 45%, respectively. The different letter in each line indicates a significant difference (*p* < 0.05, n = 3).

**Figure 5:**
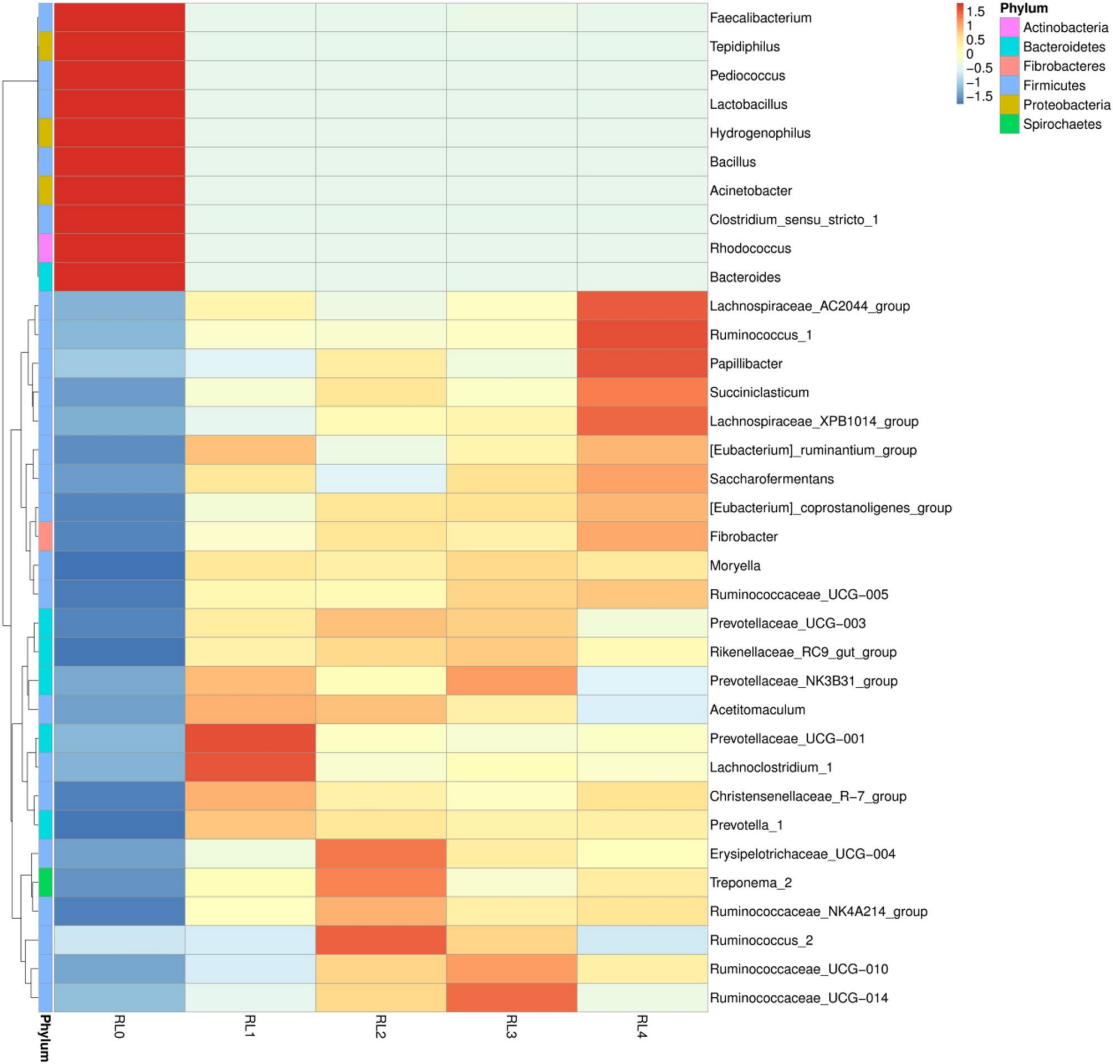
Heat map of rumen bacterial community of *Hu* sheep fed with different fermented *Pleurotus eryngii* mushroom substrate addition based on genus level. RL0 represent raw materials of fermented SMPE, RL1, RL2, RL3, RL4 represent rumen liquid of *Hu* sheep fed TMR diets contained fermented SMPE at 0%, 15%, 30% and 45%, respectively.

The relative abundance of *Lactobacillus* and *Bacteroides* in RL1, RL2, RL3 and RL4 decreased significantly compared with RL0 (*p* < 0.05), and the relative abundance of *Prevotella* 1, *Christensenellaceae* R-7, *Ruminococcaceae* NK4A214, *Fibrobacter, Rikenellaceae* RC9 and *Prevotellaceae* UCG001 increased significantly (*p* < 0.05). Compared with RL1, the relative abundance of *Christensenellaceae* R-7 in RL3 decreased significantly by 33.59%, the relative abundance of *Prevotellaceae* UCG001 in RL2, RL3 and RL4 decreased by 50.41%, 62.24% and 49.17%, respectively, and the relative abundance of *Ruminococcaceae* NK4A214 in RL2 increased significantly by 35.01% (*p* < 0.05). In addition, the analysis of bacterial community heat map based on genus level also showed that the composition of the rumen bacterial community of raw materials and the rumen liquid of *Hu* sheep with different treatments changed significantly (Fig. 5).

### Relationships between production performance and meat quality and rumen bacterial of *Hu* sheep

**Figure 6:**
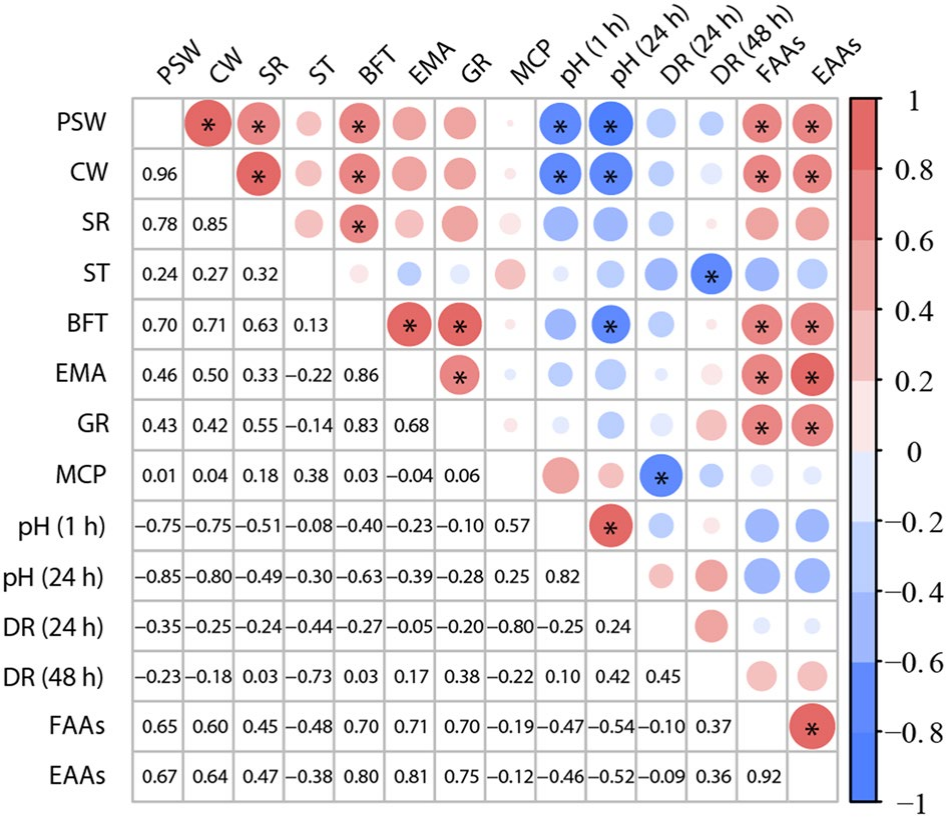
Analysis of the correlation between production performance and meat quality of *Hu* sheep. Statistical significance was calculated by Spearman’s correlation analysis (*p < 0.05). The size and intensity of the colored circles are proportional to the correlation values. PSW pre-slaughter weight, CW carcass weight, SR slaughter rate, ST skin thickness, BFT back fat thickness, EMA eye muscle area, GR grade rule, MCP meat cooking percentage, pH (1 h) and pH (24 h) = pH of the *longissimus dorsi* muscle at 1 h and 24 h after slaughter, DR (24 h) and DR (48 h) drip rate of 24 h and 48 h after slaughter, FAAs flavor amino acids, EAAs essential amino acids.

**Figure 7:**
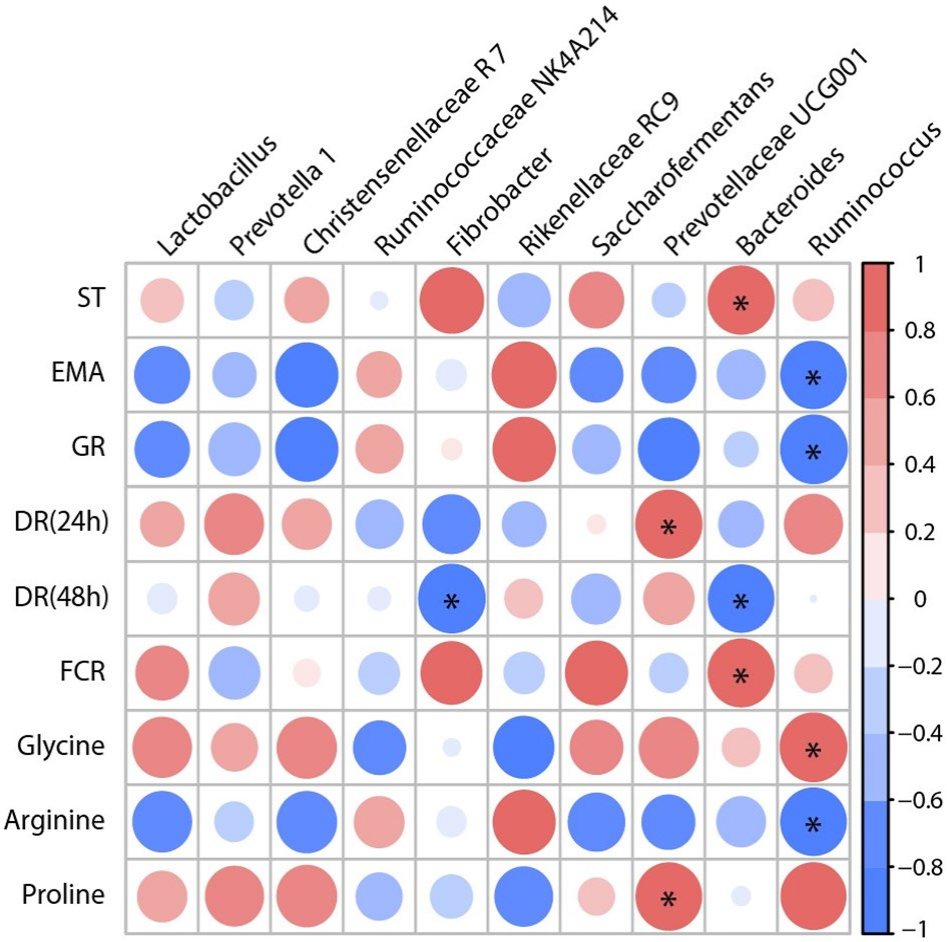
Analysis of the correlation between relative abundance of rumen bacterial based on genus level and production performance or meat quality of *Hu* sheep. Statistical significance was calculated by Spearman’s correlation analysis (*p < 0.05). The size and intensity of the colored circles are proportional to the correlation values. ST skin thickness, EMA eye muscle area, GR grade rule, DR (24 h) and DR (48 h) drip rate of 24 h and 48 h after slaughter, FCR feed conversion ratio.

To further identify potential correlations between changes in production performance and meat quality and rumen bacterial of *Hu* sheep, we calculated Spearman’s correlation coefficients between them. Firstly, we analysed the correlation between production performance and meat quality (Fig. 6). Both FAAs and EAAs showed positive correlations with PSW, CW, BFT, EMA and GR, Meanwhile, FAAs was positively correlated with EAAs. In addition, both PSW and CW were positively correlated with SR, BFT, but negatively correlated with pH (1 h) and pH (24 h), PSW was positively correlated with CW. BFT exhibited a positive correlation with SR, EMA, GR, whereas was negative correlated with pH (24 h). EMA and pH (1 h) were positively correlated with GR and pH (24 h), respectively. In contrast, DR (24 h) and DR (48 h) were negatively correlated to MCP and ST, respectively. Then, we analysed the correlation between rumen bacterial and production performance, meat quality. As shown in Fig. 7, *Prevotellaceae* UCG001 showed a positive correlation with DR (24 h) and proline. *Bacteroides* showed a positive correlation with ST and FCR and negatively correlated with DR (48 h). Similarity, *Ruminococcus* was positively correlated with glycine and negatively correlated with EMA, GR and arginine. In addition, *Fibrobacter* showed a negative correlated with DR (48 h).

## Discussion

### Effects of fermented feed on production performance and meat quality of *Hu* sheep

Production performance of animals affects the economic benefits, the improvement of animal ADG and the reduction of FCR are particularly important in the evaluation of feed value. Slaughter performance is one of the important indicators reflecting animal production performance, among which PSW, CW and SR are important factors affecting animal economic value. Gao et al*.*^4^ found that the supplementation of 30% fermented spent mushroom substrate from *Pleurotus eryngii* feed had higher body weight gain, ADG and dry matter intake, and lower FCR of *Matou* goat. Chu et al*.*^9^ showed that adding 30% fermented mushroom by‐product from *Flammulina velutipes* to the diet of growing‐fattening pigs can significantly improve the carcass weight and quality of pigs. The results of this study showed that the group fed with 15% of SMPE had high ADFI compared with RL4 and low FCR compared with RL3 and RL4. Moreover, the overall production performance of *Hu* sheep improved at 15% and 30% compared to 0% of fermented SMPE, this may be due to the beneficial bacteria and bioactive substances contained in fermented SMPE to improve the rumen environment and promote digestion of feed. Firstly, the conversion rate of mineral elements was improved in fermented SMPE inoculated with probiotics, such as LAB, *Saccharomyces cerevisiae* and *Bacillus subtilis*. Those mineral elements might combine with proteins, amino acids, polysaccharides, etc*.* in the bacteria to form as absorbable organic compounds, which had high utilization rate and biological activity^4^. Secondly, the beneficial bacteria in fermented SMPE could multiply in the rumen after eaten by animals, thus produced a variety of digestive enzymes to enhance the degradation of macromolecular substances such as fiber. Meanwhile, the endocrine secretion of digestive enzymes in the rumen were induced by the beneficial bacteria, so as to improve the conversion rate of fermented SMPE^19^, thereby improving the conversion rate of fermented feed and improving slaughter performance. Besides, the proportion of fermented SMPE should not be too high. Adding too much fermented feed will lead to a decrease in slaughter performance, which may be due to the large amount of cellulose contained in the substrate, which stimulates rumen peristalsis, accelerates the circulation rate of chyme, so that the nutrients in the chyme are not completely absorbed and are excreted, reducing the apparent digestibility of feed nutrients^20^. It can be seen that *Hu* sheep fed with 15% of fermented SMPE had the best slaughter performance.

Amino acids are important raw materials for protein synthesis, providing a material basis for host growth, metabolism and maintenance of vital signs, and their type and content are important factors affecting muscle flavor and nutritional value, and essential amino acid content is an important indicator to measure muscle quality^21^. When Boutry et al*.*^22^ fed newborn pigs a leucine-rich diet, they found that the rate of protein synthesis in the muscles of pigs was accelerated, and the signaling pathways related to protein synthesis were significantly enriched. The results of this study showed that the total amount of essential amino acids in RL2 and RL3 was significantly higher than that in RL1, indicated that the addition of 15% and 30% of fermented feed could significantly increase the amino acid content of mutton and improve the nutritional value of meat quality. Mushroom mycelium residue in the SMPE has high protein concentration, therefore, was used as a protein source for ruminants^23^. In addition, the content of essential amino acids and total amino acids of SMPE were significantly higher than those of raw materials after mixed-strain fermentation, increased by 15.45% and 25.50%, respectively^24^. The content of flavor amino acids, including aspartic acid, glutamic acid and glycine etc*.*, was higher in SMPE raw materials^24^. After fermentation, the microorganisms convert non-protein nitrogen into bacterial proteins, and some microorganisms have the function of secreting proteases, the amino acid content of SMPE is further increased, thereby increasing the amino acid content of muscle. Aspartic acid, glutamic acid and glycine are umami amino acids in muscles, and their content is an important indicator that affects the flavor of meat, of which aspartic acid and glutamic acid can also be used as drugs to treat some diseases, mainly used for the treatment of liver diseases, digestive tract diseases, encephalopathy, cardiovascular diseases, respiratory diseases and for improving muscle vitality, pediatric nutrition and detoxification. In this trial, the contents of aspartic acid, glutamic acid and glycine in RL2 and RL3 were significantly higher than those in RL1. It can be seen that the addition of 15% and 30% fermented SMPE to the diet of *Hu* sheep had a significant effect on the amino acid content in muscle, which could improve the flavor and nutritional value of mutton.

### Effects of fermented feed on the structure of rumen bacterial community in *Hu* sheep

The microorganisms in ruminant rumen mainly include bacteria, fungi and protozoa, the rumen microbial composition structure is essential for maintaining environmental homeostasis in the rumen, promoting feed digestion and absorption, and benefiting animals. The structure of rumen microbial community is affected by many factors, of which diet types, structure and feeding mode are the most important factors^25^. For example, Liu et al.^26^ found that the addition of yeast culture supplementation to diets changed the bacterial community composition of sheep’s rumen in the house feed. In this experiment, the dominant phylum of *Hu* sheep rumen was Firmicutes, Bacteroidetes and Fibrobacteres, which were basically consistent with previous studies. Previous studies have shown that the Bacteroidetes and the Firmicutes are the dominant phylum in ruminant rumens^27,28^. Evans et al*.*^29^ found that Bacteroides plays an important role in the degradation of non-fibrous substances, while Firmicutes is mainly involved in the decomposition of fibrous substances. The phylum Fibrobacteres are recognized as major bacterial degraders of lignocellulosic material in the herbivore gut^30^. After microbial fermentation, the nutritional value of SMPE was significantly improved, but due to the limitation of the raw material, the cellulose content remained at a high level. Ruminants do not produce cellulase enzymes themselves but depend on bacteria and fungi in the rumen to break down cellulose. Therefore, fed TMR diets contained fermented SMPE promote the growth and proliferation of Firmicutes and Fibrobacter in the rumen of *Hu* sheep. With the increase of the addition of fermented SMPE, the relative abundances of Firmicutes and Fibrobacteres in RL4 were significantly increased compared with those in RL1. The results showed that the addition of fermented SMPE to TMR of *Hu* sheep could promote the growth and proliferation of Firmicutes and Fibrobacter, and was conducive to the degradation of fibrous substances in the diet.

Previous studies have shown that *Prevotella* is the dominant genus of ruminant rumen^31,32^. Thoetkiattikul et al*.*^33^ discovered by high-throughput sequencing of 16S rDNA that the dominant bacteria of ruminant rumens were *Prevotella* and *Flavobacterium*. In this experiment, the dominant genera in the rumen of *Hu* sheep were *Prevotella* 1, *Christensenellaceae* R-7, *Ruminococcaceae* NK4A214, *Fibrobacter*, *Rikenellaceae* RC9, *Saccharofermentans* and *Prevotellaceae* UCG001. It was not completely consistent with the results of previous studies, and it was speculated that it might be caused by differences in animal breed, age, feed structure, feeding management, etc*. Prevotella* is considered to be a degrading genus with a strong ability to decompose hemicellulose^34^, and plays an important role in the degradation of crude proteins, starches, xylan, and pectin^33,35^. The results of this experiment showed that the relative abundance of *Prevotella* 1 in the rumen of *Hu* sheep was the highest, which was consistent with the previous results, but the difference between the groups was not significant. This may be due to the fact that the diet designed for this study had similar crude protein and energy levels, so it did not have a significant effect on the relative abundance of *Prevotella*. *Ruminococcaceae* include *ruminococcus flavefaciens* and *ruminococcus albus* and are the main fibro degrading bacteria in the rumen and can produce a large amount of cellulase, hemicellulase and xylanase to degrade cellulose and hemicellulose in forage. In this study, the relative abundance of *Ruminococcaceae* NK4A214 in RL2 was significantly higher than RL1. The results showed that the addition of fermented SMPE to TMR of *Hu* sheep could promote the growth and reproduction of rumen and improve the degradation rate of fiber in the diet.

Diet plays an important role in shaping the rumen microbes in ruminants, alters the rumen's environment and metabolism^36,37^. Then alters the muscle metabolism and meat quality. For example, TMR containing palm kernel meal at 18% of Tibetan sheep increased the abundance of *Christensenellaceae* R-7, *Ruminococcaceae* UCG-013, *Lachnospiraceae* UCG-002, and *Family XIII AD3011* in the rumen but decreased the abundance of *Prevotella* 1, the above bacteria groups regulate meat quality by regulating rumen metabolites (succinic acid, DL-glutamic acid, etc.)^38^. In this study, RL2, RL3 and RL4 decreased the abundance of *Prevotellaceae* UCG001 compared with RL1. Correlation analysis shown that *Prevotellaceae* UCG001 was positive correlation with DR (24 h) and proline. Indicated that the addition of fermented SMPE to diets may regulate meat quality of *Hu* sheep by affecting microorganisms in the rumen, since rumen microbes might affect the production of VFA and microbial protein by microbial fermentation of feedstuff^39^, the above functional metabolites subsequently alters the deposition of metabolites in muscles. Therefore, further research is needed to measure the end products of the fermentation process to determine how SMPE feed alters internal environment homeostasis and flora composition in the rumen to affect meat quality.

## Materials and methods

### Preparation of spent mushroom substrate from *Pleurotus eryngii*

SMPE was provided by Fujian Greenbao Group, the cultivation material formula was as follows: bagasse 13.0%, wood chips 22.2%, corn cob 26.0%, bran 18.0%, corn flour 8.8%, soybean meal 9.0%, lime 1.2%, light calcium 1.8%, which was adapted from previously described^40^. Waste fungus sticks were selected after harvest the mushrooms for 1 time, the mycelium was white, fresh and mildew-free, then unbagged, crushed and set aside.

The compound microbial agent “Huojunduo” was bought from Beijing Shengyuda Biotechnology Co., Ltd. Number of viable bacteria: *Bacillus subtilis* ≥ 100 × 10^6^ CFU·g^−1^, lactic acid bacteria ≥ 10 × 10^6^ CFU·g^−1^, *Saccharomyces cerevisiae* ≥ 100 × 10^6^ CFU·g^−1^, total bacterial ≥ 210 × 10^6^ CFU·g^−1^. The formula of spent mushroom substrate mixed fermentation material was: 500 kg of fungus bran, 260 kg of fine bran, 180 kg of barley, 10 kg of brown sugar, 1 kg of fermentation fungus agent and 50 kg of water, which was adapted from the method as described by Gao et al^10^. The microbial agent was evenly sprayed on the raw materials, mixed evenly and divided into sealed bags, and fermented anaerobically at room temperature for 21 days. The nutrients before and after fermentation were shown in Table S.3.

### Experimental design

The experiment was conducted at Longyan Green Tao Animal Husbandry Co., Ltd. in Gaopi Town, Yongding District, Longyan City, Fujian Province, China (24.96◦ N, 116.86◦ E, above sea level 310 m). The present study used 120 *Hu*-breed lambs, the ratio of male to female stands at 1:1, around 60 days of age, the mean body weight was 13.50 kg (SD = 3.10) at the start of the performance test, all the male sheep were castrated. A univariate experimental design was used and randomized into 4 groups with 3 replicates in each group and 10 *Hu* sheep per replicate. 0 (RL1), 15% (RL2), 30% (RL3) and 45% (RL4) of fermented SMPE were added to TMR respectively, and the diet formula and nutrient level were shown in Table S.4. The trial was carried out by house feeding, sheep were adapted to the diet for a period of 10 days followed by 150 days of growth performance recording. Before the test, the sheep were numbered and dewormed, the sheep house was cleaned regularly, disinfected on time, and raised by special personnel in the well-ventilated sheep house. Feed once a day at 8:00 and 17:00 and drink freely. Feed should be based on a slight surplus in the trough, and the surplus should be collected and measured daily.

### Measurement indicators and method

#### Chemical composition analysis

The samples were dried in a forced-air oven at 60 °C for 72 h and then ground and sieved through a 1 mm sieve for chemical analysis. Moisture, crude ash, crude fibre (CF), and crude protein (CP) contents were determined using the method from the Association of Official Analytical Chemists^41^. Neutral detergent fibre (NDF), acid detergent fibre (ADF) contents were determined using the procedure reported by Van Soest et al.^42^. Calcium and total phosphorus were determined by the potassium permanganate method and the ammonium molybdate spectrophotometric method, respectively^41^. Metabolizable energy were calculated according to the method of Gao et al.^43^.

#### Production performance determination

Average daily feed intake (*ADFI*) was measured based on the difference between the offered and residual feed. The body weight of individual animals was measured weekly, and the weight gain (*WG*) was calculated based on the difference between the initial body weight (*IBW*) and final body weight (*FBW*). The feed conversion ratio (*FCR*) was determined cumulatively through the collected data.

At the end of the experimental, 3 test male sheep were randomly selected in each group (1 sheep per replicate), fasting for 24 h and water for 2 h before slaughter. Slaughter before weighing, cut neck and bleed, peel off fur, remove head, hooves and internal organs, and measure slaughter performance indicators, including carcass weight (*CW*), slaughter rate (*SR*), skin thickness (*ST*), eye muscle area (*EMA*), back fat thickness (*BFT*), grade rule (*GR*), etc*.* CW: pre-slaughter live weight to remove the weight of the head, fur, hooves, tail, and internal organs (retaining the kidneys and surrounding fat); SR(%) = CW/ weight before slaughter × 100; EMA: the cross-sectional area of the superior spine-ophthalmic muscle between the 12th and 13th ribs, the cross-sectional contour of the eye muscle was depicted with sulfuric acid paper, and then the contour area was calculated by the accumulation meter (QCJ-2000, Shandong); GR value: The thickness of the tissue between the 12th and 13th ribs was measured 11 cm from the midline of the dorsal spine with a vernier caliper.

#### Meat quality determination

The left *longissimus dorsi* muscle was taken after slaughtered to determine the meat quality indicators such as meat cooking percentage (*MCP*), pH and drip rate (*DR*). MCP: peel off the muscle-attached fat, weigh with a balance with a sensitivity of 0.1 g (count as W1), put the meat sample into a polyethylene packaging bag, steam in an aluminum steamer for 30 min, take it out, and put in cool water to cool down for 1 h, dry the surface moisture with paper, weigh (count as W2), MCP(%) = W2/W1 × 100%. pH: The pH was measured 45 min after slaughter with a pH meter, and the average value was taken as the final result. DR: Cut 2 pieces of meat with a length of 5 cm, width of 3 cm and thickness of 2 cm, put them in plastic bags (meat samples must not contact the wall of plastic bags), tie the mouth of the bag tightly and hang them in a refrigerator at 4 °C, take them out after 24 h and 48 h, absorb the moisture on the surface with absorbent paper and weigh, record as the final weight. DR (%) = (initial weight－final weight) / initial weight × 100%. The amino acid composition of the *longissimus dorsi* muscle was determined. After treatment with a freeze dryer before testing, smashed and passed through a 0.425 mm sieve. Referring to the method of GB 5009.168-2016, the Hitachi L-8900 automatic amino acid analyzer (Japan) was used for measurement.

### Sample determination and data analysis

#### DNA extraction and PCR amplification of rumen liquid

After slaughter, the rumen contents were immediately collected from the rumen of 3 sheep in each treatment, transferred to sterile containers, stored in dry ice, and transported to the laboratory at − 80 °C for DNA extraction. The total DNA of the microbial genome of the samples was extracted by CTAB. DNA concentration was determined by Nanodrop and DNA quality was detected by 1.2% agarose gel electrophoresis. Dilute DNA to 1 ng·μl^−1^ using sterile water. The V4 region of the bacterial 16S rDNA gene was amplified using universal primers 515F (5′-GTGCCAGCMGCCGCGGTAA-3′) and 806R (5′-GGACTACHVGGGTWTCTAAT-3′). PCR products were detected using a 2.0% agarose gel for electrophoresis and bands of interest were recovered by the Gene JET Gel Recovery Kit (Thermo Scientific). The recovered products were sent to the IonS5^TM^XL platform of Beijing Novogene Technology Co., Ltd. for high-throughput sequencing.

#### Statistical analysis

After the sequencing data were quality controlled by Fast QC, Cutadapt (V1.9.1) was used to filter short sequences (< 200 bp) and low-quality sequences (*q* < 25). Operational Taxonomic Units (OTUs) are assigned to valid sequences with 97% similarity with the SILVA database by the Mothur method. The sample diversity index was calculated using Qiime (Version 1.9.1) and principal component analysis was performed using R.

All experiments were performed in triplicate and the data obtained were expressed as mean ± standard deviation (SD). For determinations of growth performance, slaughter performance, amino acid content of mutton, meat quality, relative abundance and diversity index of rumen bacterial community of *Hu* sheep, one-way analysis of variance (ANOVA) was done followed by Fisher’s least significant difference (LSD) at *p* = 0.05 using SPSS 22.0 for Windows (SPSS Inc, Chicago, USA).

To identify putative correlations between production performance and meat quality and rumen microbial composition of *Hu* sheep, Spearman’s rank correlation coefficients for pairwise comparisons were calculated and visualized with the corrplot package in R.

## Conclusion

Overall, the production performance of *Hu* sheep was superior while fed with TMR contained fermented SMPE at 15% relative to the other groups. RL2 and RL3 resulted in an increase in EAAs and FAAs content. For rumen bacteria, the relative abundances of Firmicutes and Fibrobacteres increased in RL4, and *Ruminococcaceae* NK4A214 increased in RL2. However, RL2, RL3 and RL4 decreased the abundance of *Prevotellaceae* UCG001 compared with RL1. The correlation analysis indicated that the addition of fermented SMPE to TMR may improve the production performance and meat quality by affecting microorganisms in the rumen of *Hu* sheep.

## Ethics statement

All of the procedures involving animals were approved by the Animal Care and Use Committee and Ethics Committee at the Agriculture Ecology Research Institute, Fujian Academy of Agricultural Sciences (NO. PZCASFAAS21022). We obtained written informed consent to use the animals in this study from the owners of the animals. All of the experiments were performed in accordance with the recommendations in the Regulations for the Administration of Affairs Concerning Experimental Animals of the State Council of the People’s Republic of China. All of the experiments were carried out in compliance with the ARRIVE guidelines.

## Supplementary Information

Supplementary Information.

## Data Availability

The raw datasets generated during the current study are available in the NCBI (PRJNA944903) repository.
