# Supplementary material for: Production performance and rumen bacterial community structure of Hu sheep fed fermented spent mushroom substrate from Pleurotus eryngii
Source: Sci Rep. 2023 May 29;13:8696. doi: 10.1038/s41598-023-35828-8 (PMC10227014; doi:10.1038/s41598-023-35828-8)
Supplement: Supplementary file 1 — Supplementary Information. [file 41598_2023_35828_MOESM1_ESM.docx]

**Production performance and rumen bacterial community structure of *Hu* sheep fed fermented** **spent mushroom substrate from *Pleurotus eryngii***

^+^ Xiaoyun Huang^1,2^, ^+^ Liuting Zhou^1,2^, Xiaofeng You^1,2^,

Haidong Han^1,2^, Xinzhu Chen^3*^, Xiusheng Huang^1,2*^

**Table S.1. Effects of different fermented *Pleurotus eryngii* mushroom substrate addition on meat quality of *Hu* sheep**

| Treatment | MCP（%） | pH（1 h） | pH（24 h） | DR（%，24 h） | DR（%，48h） |
| --- | --- | --- | --- | --- | --- |
| RL1 | 57.41±0.88 | 6.23±0.30 | 6.14±0.27 | 1.30±0.59 | 1.73±0.30 |
| RL2 | 55.69±0.40 | 5.99±0.11 | 5.88±0.05 | 1.14±0.34 | 1.73±0.58 |
| RL3 | 59.66±0.22 | 6.42±0.51 | 6.03±0.07 | 0.79±0.18 | 1.89±0.75 |
| RL4 | 58.78±2.81 | 6.13±0.06 | 6.00±0.04 | 0.82±0.39 | 1.31±0.01 |

Note: RL1, RL2, RL3, RL4 represent groups of *Hu* sheep fed TMR diets contained fermented SMPE at 0%, 15%, 30% and 45%, respectively. MCP = meat cooking percentage, DR = drip rate. A different letter in each column indicates a significant difference (*p*<0.05，n=3).

**Table S.2 The α diversity index of rumen bacterial community of *Hu* sheep fed with different fermented *Pleurotus eryngii* mushroom substrate addition**

| Items | Observed species | Shannon index | Simpson index | Chao1 index | ACE index |
| --- | --- | --- | --- | --- | --- |
| RL0 | 377.00±179.16b | 3.23±3.00b | 0.50±0.40b | 432.65±191.05b | 414.28±193.31b |
| RL1 | 945.00±15.56a | 7.06±0.24a | 0.97±0.01a | 1136.76±196.91a | 1105.47±130.84a |
| RL2 | 991.00±93.34a | 7.32±0.25a | 0.98±0.00a | 1067.60±101.55a | 1076.94±96.76a |
| RL3 | 1083.00±33.94a | 7.38±0.12a | 0.98±0.00a | 1140.77±29.31a | 1160.95±29.60a |
| RL4 | 983.00±19.80a | 7.09±0.43a | 0.97±0.01a | 1070.23±25.21a | 1072.77±29.50a |

Note：RL0 represent raw materials of fermented SMPE, RL1, RL2, RL3, RL4 represent rumen liquid of *Hu* sheep fed TMR diets contained fermented SMPE at 0%, 15%, 30% and 45%, respectively. A different letter in each column indicates a significant difference (*p*<0.05，n=3).

**Table S.3 Dry matter nutrients of raw materials and mixed fermentation materials of spent mushroom substrate from *Pleurotus eryngii* (%)**

| **Items** | **Raw materials** | **Mixed fermentation materials** |
| --- | --- | --- |
| Moisture | 55.45 | 46.79 |
| CP | 6.16 | 6.55 |
| CF | 19.45 | 31.75 |
| NDF | 70.92 | 59.98 |
| ADF | 59.07 | 44.09 |
| CA | 10.93 | 9.43 |

Note: CP = crude protein, CF = crude fiber, NDF = neutral detergent fiber, ADF = acid detergent fiber, CA = crude ash.

**Table S.4 Diet formula and nutritional level of *Hu* sheep (Dry matter base)**

| **Items** | **Group** | | | |
| --- | --- | --- | --- | --- |
|  | **RL1** | **RL2** | **RL3** | **RL4** |
| Fermented mushroom substrate(%) | 0 | 15 | 30 | 45 |
| Peanut seedlings(%) | 45 | 32 | 19 | 8 |
| Corn(%) | 25 | 21 | 18 | 14 |
| Wheat bran(%) | 12.5 | 14.5 | 15.5 | 15.5 |
| Soybean meal (%) | 11 | 11 | 11 | 11 |
| Dicalcium phosphate(%) | 1 | 1 | 1 | 1 |
| Salt(%) | 0.5 | 0.5 | 0.5 | 0.5 |
| Premix(%) | 5 | 5 | 5 | 5 |
| Total(%) | 100 | 100 | 100 | 100 |
| Nutritional level | | | | |
| CP(%) | 14.62 | 14.38 | 14.24 | 14.06 |
| NDF(%) | 35.79 | 36.85 | 37.86 | 38.04 |
| ADF(%) | 28.42 | 29.33 | 30.57 | 31.53 |
| Calcium(%) | 1.44 | 1.48 | 1.52 | 1.54 |
| Phosphorus(%) | 0.5 | 0.54 | 0.56 | 0.58 |
| Metabolic energy(MJ·kg^-1^） | 9.65 | 9.71 | 9.74 | 9.77 |

Note: RL1, RL2, RL3, RL4 represent groups of *Hu* sheep fed TMR diets contained fermented SMPE at 0%, 15%, 30% and 45%, respectively. CP = crude protein, NDF = neutral detergent fiber, ADF = acid detergent fiber. Per kilogram of premix contain Vitamin A>100 000 IU; Vitamin D_3_ 40 000~80 000 IU; Vitamin E>700 IU; Iron 1.2~3.5 g; Copper 0.3~0.9 g; Manganese 1~3 g; Zinc 2.0~5.5 g; Iodine 20~60 mg; Selenium 10~30 mg; Drill 15~45 mg; Calcium 8.5%~20%; Total phosphorus >3.5%; Sodium chloride 20%~30%.


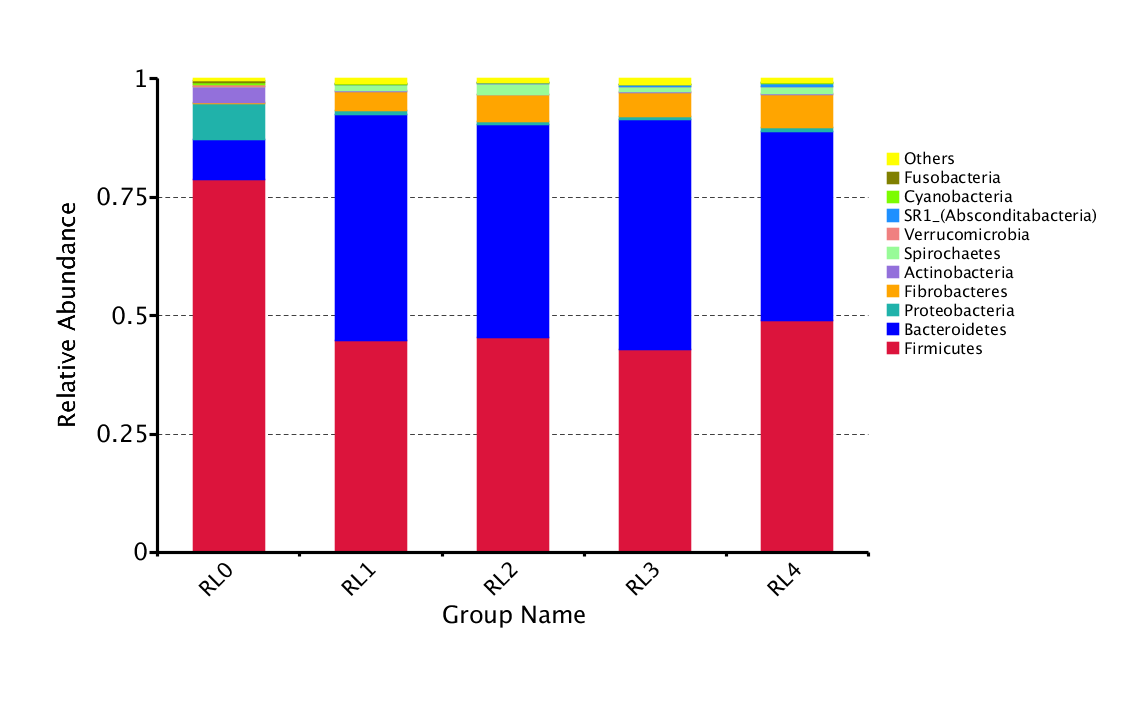


**Figure S.1 Relative abundance of rumen bacterial community of *Hu* sheep fed with different fermented *Pleurotus eryngii* mushroom substrate addition based on phylum level. RL0 represent raw materials of fermented SMPE, RL1, RL2, RL3, RL4 represent rumen liquid of *Hu* sheep fed TMR diets contained fermented SMPE at 0%, 15%, 30% and 45%, respectively.**
